# Microbiome dataset of bacterial and fungal communities in anthosphere of twelve different wild plants in South Korea

**DOI:** 10.1016/j.dib.2023.109470

**Published:** 2023-08-05

**Authors:** Jihoon Kim, Yingshun Cui, Haeun Lee, Seong-Jun Chun

**Affiliations:** a LMO Team, National Institute of Ecology, 1210 Geumgang-ro, Maseo-myeon, Seocheon 33657, Republic of Korea; b Protected Area Research Team, National Institute of Ecology, 1210 Geumgang-ro, Maseo-myeon, Seocheon 33657, Republic of Korea; c LED Agri-bio Fusion Technology Research Center, Jeonbuk National University, 79 Gobong-ro, Iksan, Republic of Korea

**Keywords:** Floral microbiome, Bacterial community, Fungal community, Anthosphere

## Abstract

This dataset provides detailed profiles of bacterial and fungal communities associated with flowers (anthosphere) of 12 different plant species collected from remote and secluded locations characterized by a flourishing and diverse plant ecosystem. In total, 144 flower samples were collected from 12 different wild plants. Bacterial 16S rRNA and fungal ITS genes obtained using the Illumina Miseq approach were used to describe the anthosphere. Metadata and raw sequences obtained in this study are available from the National Center for Biotechnology Information (BioProject ID: PRJNA983070). Amplicon Sequence Variants (ASVs) of bacteria and fungi were analyzed using the DADA2 pipeline. After quality filtering, trimming, and removing the chimeric sequences, 2076 bacterial and 2152 fungal ASVs were identified in the anthosphere. Burkholderiales and Enterobacterales in bacteria, and Pleosporales in fungi were the predominant groups in the anthosphere regardless of the plant species. Among the twelve different plant species, *Forsythia koreana* exhibited the highest abundance of both bacterial and fungal groups. This dataset represents a detailed exploration of the anthosphere in the most abundant and commonly observed plant species in South Korea, and provides new insights into the microbial communities and interactions of the anthosphere.

**Specifications Table**

**Table:** 

Subject	Environmental genomics and metagenomics
Specific subject area	16S rRNA and ITS metagenomics of wild plant flowers.
Type of data	Amplicon sequencing data of 16S rRNA and ITS region
How the data were acquired	DNA sequences: Illumina Miseq platform Data processing: DADA2 v. 1.16.0. Data analysis: R v. 4.1.2.
Data format	Raw, filtered, and analyzed
Type of data	Amplicon sequencing data of 16S rRNA and Internal Transcripted Spacer (ITS) region
Data collection	To explore anthosphere, a total of 144 different flower samples were collected from twelve different wild plant species, *Brassica juncea* (brown mustard), *Veronica polita* (grey field-speedwell), *Capsella bursa-pastoris* (shepherd's purse), *Lamium amplexicaule* (common henbit), *Lamium purpureum* (red dead-nettle), *Taraxacum platycarpum* (Korean dandelion), *Duchesnea indica* (mock strawberry), *Forsythia koreana* (Korean goldenbell tree), *Vicia villosa* (hairy vetch), *Chelidonium majus* var. *asiaticum* (greater celandine), *Prunus jamasakura* (Japanese cherry), and *Viola mandshurica* (violet) in a remote and secluded location characterized by a flourishing and diverse plant ecosystem. ASVs of bacterial 16S rRNA and fungal ITS genes were targeted to identify microbial communities in anthosphere.
Data source location	-Institution: National Institute of Ecology -Sampling site: Gurye, Jeollanam-do -Country: Republic of Korea -Sampling date: April 8, 2022 -Latitude and longitude: 35.19°N, 127.47°E
Data accessibility	Raw sequences Repository name: NCBI SRA Data identification number: PRJNA983070 Direct URL to data: https://www.ncbi.nlm.nih.gov/bioproject/983070 Accessions: SAMN35719144-SAMN35719155

## 1 Value of the Data

• The bacterial and fungal microbiome datasets provided the variation patterns of microbial community structures harbored in different flowering plants that faced similar environmental and ecological conditions.
• This dataset is valuable for understanding interactions within anthosphere microbes in South Korea.
• This is the first study to explore the anthospheres of *Veronica polita, Taraxacum platycarpum, Forsythia koreana, Chelidonium majus* var. *asiaticum, Prunus jamasakura,* and *Viola mandshurica*.
• This dataset can be used as a comparative dataset for scientists interested in exploring the differences among flowering plants in different regions and countries.

## 2 Objective

The plant microbiome plays a pivotal role in plant physiology. Most plant microbiome studies have focused on the rhizosphere to uncover complex interactions between plants and microbes [1]. The anthosphere, one of the most important parts of the phyllosphere, has recently been increasingly used to explore flower-related microbial roles and their influence on plant physiology [2]. Anthosphere microbes influence the phenotype of flowers and affect their interactions with pollinators [3]. In this study, we collected 144 different flower samples from twelve different plant species that faced similar environmental and ecological conditions and analyzed both bacteria and fungi to identify the microbial variations in anthosphere and hidden interspecies interactions in relation to the different plant species.

## 3 Data Description

**Fig. 1:**
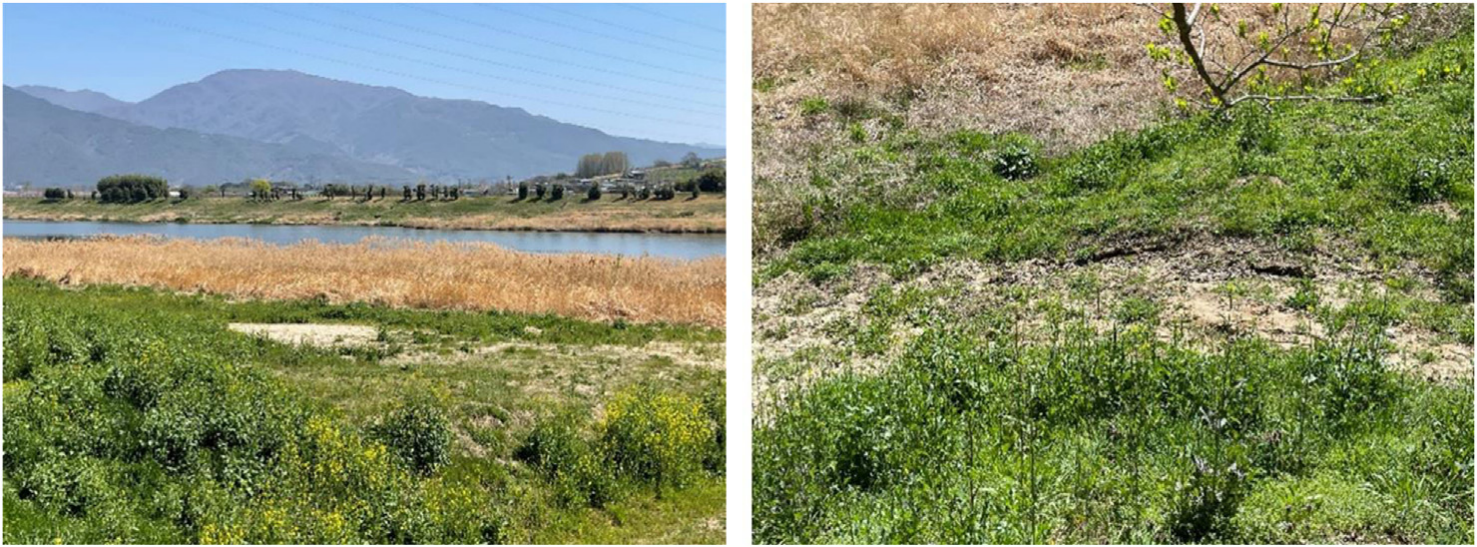
Landscape of the sampling site and sampled flowers.

**Fig. 2:**
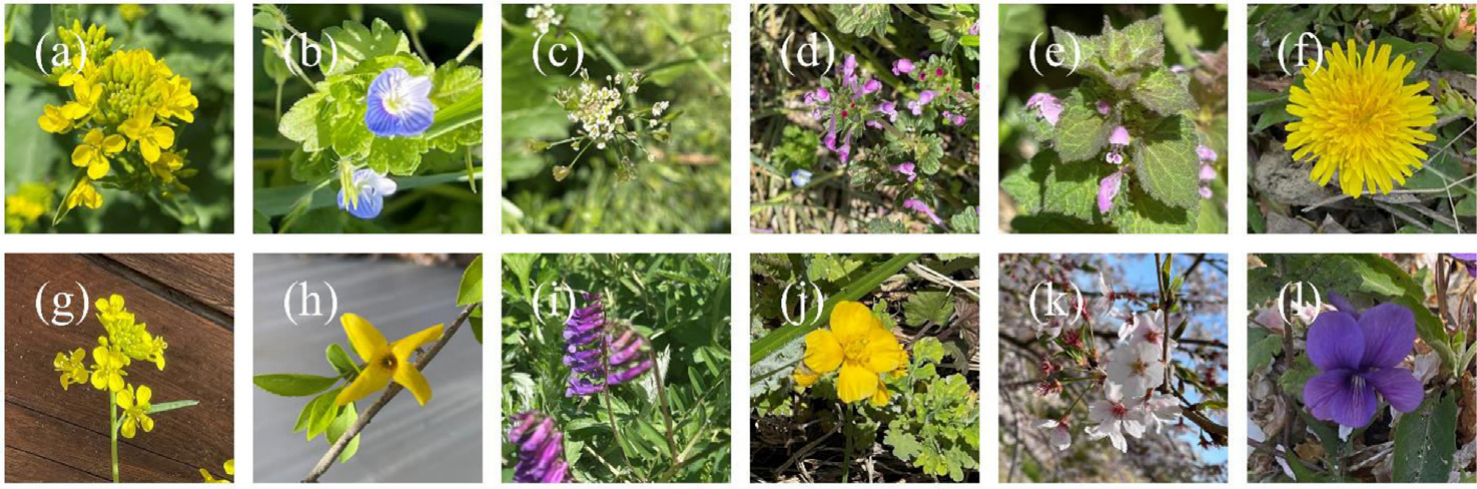
Pictures of the collected samples. (a) *Brassica juncea*, (b) *Veronica polita*, (c) *Capsella bursa-pastoris*, (d) *Lamium amplexicaule*, (e) *Lamium purpureum*, (f) *Taraxacum platycarpum*, (g) *Duchesnea indica*, (h) *Forsythia koreana*, (i) *Vicia villosa*, (j) *Chelidonium majus* var. *asiaticum*, (k) *Prunus jamasakura*, and (l) *Viola mandshurica*.

**Fig. 3:**
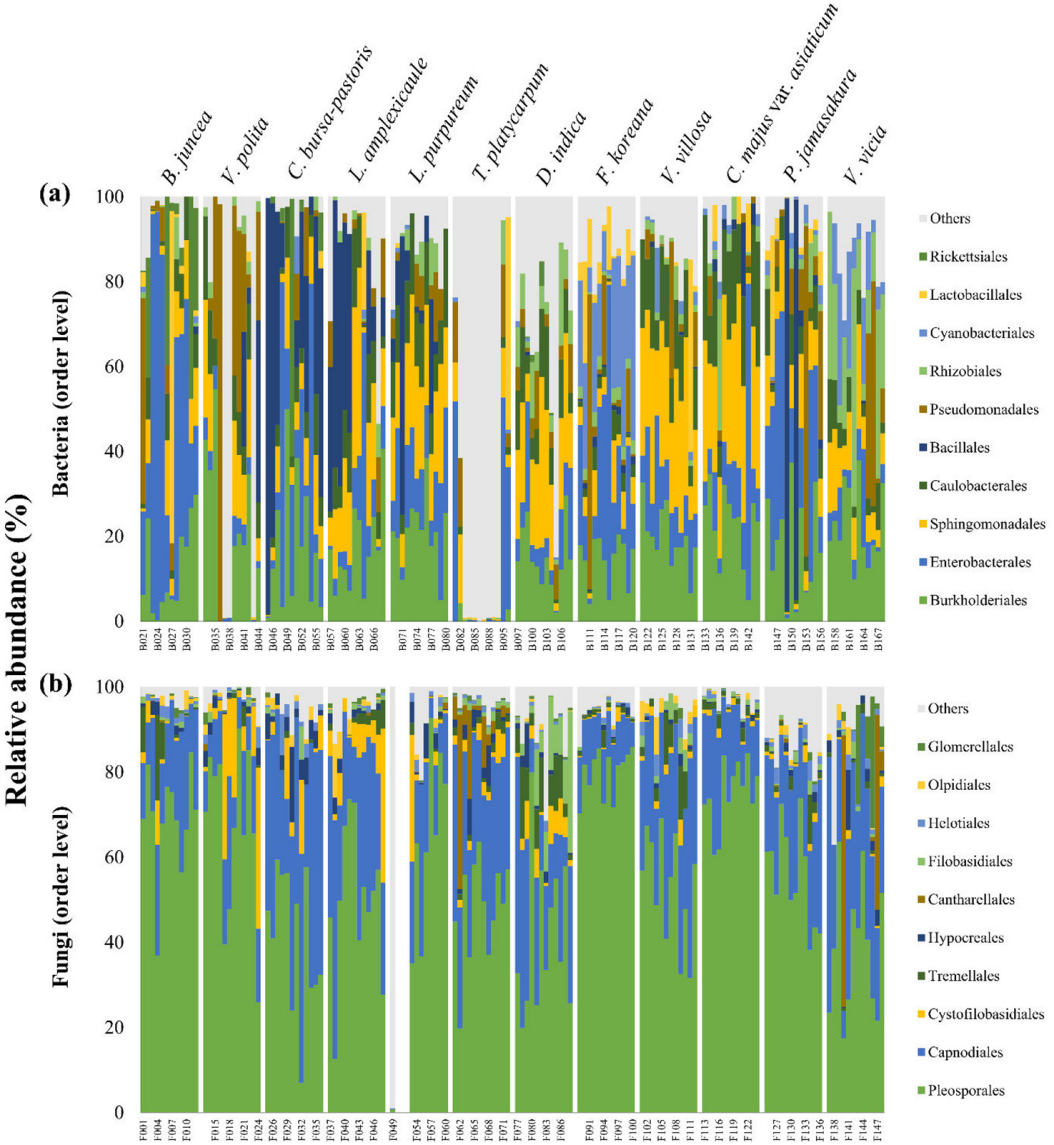
Bar plots showing the relative abundance of bacteria and fungi. The relative abundance of (a) bacterial and (b) fungal communities in flower samples of twelve different plant species at the order level.

Flower samples were collected from Gurye (35.19°N, 127.47°E), South Korea in April 2023 (Figs. 1 and 2). Both bacterial and fungal microbiomes were analyzed using the Illumina Miseq approach. After filtering, trimming, and removing chimeric contaminants, 2076 bacterial ASVs (total read: 567,462) and 2152 fungal ASVs (total read: 3,531,269) were identified and used for further analysis (Table 1). The top five most abundant bacterial groups were Burkholderiales (18.13% ± 11.07%), Enterobacterales (17.50% ± 20.80%), Sphingomonadales (15.24% ± 11.00%), Caulobacterales (8.98% ± 6.87%), and Bacillales (8.69%±20.44%) at the order level (Fig. 3a). Pleosporales and Capnodiales fungi accounted for an average of 81.14 ± 13.39% in total fungal community at the order level (Fig. 3b), indicating flower-specific fungal adaptations could be more uniform compared to bacterial groups in anthosphere. Regardless of the plant species, Burkholderiales and Enterobacterales in bacteria and Pleosporales in fungi were the dominant groups in the anthosphere. In addition, the diversity of anthospheric microbes varies among plant species. *Forsythia koreana* harbored the highest bacterial and fungal species (101.08 ± 22.33 bacterial ASVs and 65.33 ± 17.62 fungal ASVs), while *Taraxacum platycarpum* and *Vicia villosa* harbored the lowest bacterial (7.67 ± 7.23 ASVs) and fungal (24.58 ± 7.67 ASVs) species, respectively.

**Table 1:** Number of ASVs of bacteria and fungi observed in anthosphere of different plant species.

Plant species	Number of bacterial ASVs (samples)	Number of fungal ASVs (samples)
Brassica juncea	26.58 ± 24.53 (n = 12)	43.58 ± 14.69 (n = 12)
Veronica polita	14.67 ± 11.86 (n = 12)	35.08 ± 10.89 (n = 12)
Capsella bursa-pastoris	16.33 ± 10.24 (n = 12)	33.50 ± 10.29 (n = 12)
Lamium amplexicaule	33.67 ± 24.88 (n = 12)	40.75 ± 11.05 (n = 12)
Lamium purpureum	23.92 ± 6.91 (n = 12)	34.33 ± 10.32 (n = 9)
Taraxacum platycarpum	7.67 ± 7.23 (n = 12)	58.25 ± 13.88 (n = 12)
Duchesnea indica	38.58 ± 14.41 (n = 12)	55.50 ± 19.56 (n = 12)
Forsythia koreana	101.08 ± 22.33 (n = 12)	65.33 ± 17.62 (n = 12)
Vicia villosa	31.92 ± 10.14 (n = 12)	24.58 ± 7.67 (n = 12)
Chelidonium majus var. asiaticum	22.67 ± 15.29 (n = 12)	28.58 ± 9.24 (n = 12)
Prunus jamasakura	25.67 ± 9.68 (n = 12)	56.17 ± 11.74 (n = 12)
Viola mandshurica	41.50 ± 16.90 (n = 12)	36.25 ± 13.32 (n = 12)

## 4 Experimental Design, Materials and Methods

### 4.1 Study site and sampling design

Flower sampling was conducted on April 8, 2022, in Gurye (35.19°N, 127.47°E), South Korea. A total of 144 samples were obtained from a diverse range of twelve distinct plant species, with twelve flower samples obtained from each plant species. The sampling site was selected based on its abundance and diversity of wild plant species. To minimize within-species contamination of different plant types, all tools were sterilized with 80% ethanol before collecting the flower samples. After collecting the samples, each flower was stored in 1.7 mL e-tubes separately and immediately placed on dry ice until further analysis.

### 4.2 DNA extraction and sequencing

DNA was extracted using the FastDNA™ SPIN Kit for Soil (MP Biomedicals, CA, USA) according to the manufacturer's instructions and quantified using a Nanodrop 2000 UV spectrophotometer (Thermo Scientific, DE, USA). Bacterial 16S rRNA gene was amplified by using a universal primer set of 341F (5′-CCTACGGGNGGCWGCAG-3’) and 805R (5′-GACTACHVGGGTATCTAATCC-3’), which targeting V3-V4 region of bacterial 16S rRNA gene [4]. The fungal ITS1 region was amplified using the fungal universal primer sets ITS1F_KYO1 (5′- CTHGGTCATTTAGAGGAASTAA-3’) and ITS2_KYO2 (5′- TTYRCTRCGTTCTTCATC-3’) [5]. Dual-PCR amplification, purification, and quantification were performed according to the Illumina 16S metagenomic sequencing library protocol described previously [6]. Distilled water was used as a negative control. The final products were used for paired-end read sequencing reactions and sequenced using MiSeq (2  ×  300 bp reads) obtained from Macrogen Corporation (Seoul, South Korea).

### 4.3 Bioinformatic analysis

To investigate the ASVs profiles of different flowers, the ASVs of bacterial 16S rRNA and fungal ITS genes were calculated using DADA2 (version 1.16.0) according to the pipeline tutorial (1.16) (https://benjjneb.github.io/dada2/tutorial.html) and DADA2 ITS Pipeline Workflow (1.8) (https://benjjneb.github.io/dada2/ITS_workflow.html), respectively [7]. Singletons, doubletons, and tripletons were removed from the 16S rRNA and fungal ITS gene datasets before analysis. The latest Silva database (release 138.1) [8] and the latest UNITE general FASTA release for fungi (version 9.0) [9] were used to classify bacterial and fungal sequences, respectively. Any reads identified as chloroplast, mitochondrial, and negative control sequences were excluded from the 16S rRNA database. Three fungal samples were omitted from the data analyses because they did not satisfy the quality control thresholds.

## Ethics Statement

The authors have read and followed the ethical requirements for publication in *Data in Brief* and confirm that the current work does not involve human subjects, animals experiments or any data collected from social media platforms.

## CRediT authorship contribution statement

**Jihoon Kim:** Investigation, Methodology, Visualization, Writing – original draft. **Yingshun Cui:** Investigation, Writing – review & editing. **Haeun Lee:** Investigation, Visualization. **Seong-Jun Chun:** Conceptualization, Investigation, Writing – review & editing, Supervision.

## Data Availability

Floral microbial community structure of 12 wild plants in Gurye, Korea Republic (Original data) (NCBI SRA).
